# Diaphragmatic CMAP Monitoring During Cryoballoon Procedures: Surface vs. Hepatic Recording Comparison and Limitations of This Approach

**DOI:** 10.3389/fcvm.2022.814026

**Published:** 2022-02-08

**Authors:** Lilith Tovmassian, Baptiste Maille, Linda Koutbi, Jérôme Hourdain, Elisa Martinez, Maxime Zabern, Jean-Claude Deharo, Frédéric Franceschi

**Affiliations:** ^1^Department of Cardiology, CHU Timone, Marseille, France; ^2^Aix-Marseille Université, Faculté de Médecine,Marseille, France; ^3^Center for CardioVascular and Nutrition Research (C2VN), INSERM, INRA, Marseille, France; ^4^Aix-Marseille University, Marseille, France

**Keywords:** atrial fibrillation, pulmonary vein isolation, cryoballoon ablation, phrenic nerve palsy, compound motor action potential

## Abstract

**Background:**

Compound motor action potential (CMAP) monitoring is a common method used to prevent right phrenic nerve palsy during cryoballoon ablation for atrial fibrillation.

**Objective:**

We compared recordings simultaneously obtained with surface and hepatic electrodes.

**Methods:**

We included 114 consecutive patients (mean age 61.7 ± 10.9 years) admitted to our department for cryoballoon ablation. CMAP was monitored simultaneously with a hepatic catheter and a modified lead I ECG, whilst right phrenic nerve was paced before (stage 1) and during (stage 2) the right-sided freezes. If phrenic threat was detected with hepatic recordings (CMAP amplitude drop >30%) the application was discontinued with forced deflation.

**Results:**

The ratio of CMAP/QRS was 4.63 (2.67–9.46) for hepatic and 0.76 (0.55–1.14) for surface (*p* < 0.0001). Signal coefficients of variation during stage 1 were 3.92% (2.48–6.74) and 4.10% (2.85–5.96) (*p* = 0.2177), respectively. Uninterpretable signals were more frequent on surface (median 10 vs. 0; *p* < 0.0001). For the 14 phrenic threats, the CMAP amplitude dropped by 35.61 ± 8.27% on hepatic signal and by 33.42 ± 11.58% concomitantly on surface (*p* = 0.5417). Our main limitation was to achieve to obtain stable phrenic capture (57%). CMAP monitoring was not reliable because of pacing instability in 15 patients (13.16%). A palsy occurred in 4 patients (3.51%) because cryoapplication was halted too late.

**Conclusion:**

Both methods are feasible with the same signal stability and amplitude drop precocity during phrenic threats. Clarity and legibility are significantly better with hepatic recording (sharper signals, less far-field QRS). The two main limitations were pacing instability and delay between 30% CMAP decrease and cryoapplication discontinuation.

## Introduction

Cryoballoon pulmonary vein isolation (PVI) is a well-established, effective and safe alternative to radiofrequency (1–5) for atrial fibrillation (AF) treatment. Although it is a highly standardized and reproducible procedure, it remains associated with a significant risk of transient or permanent phrenic nerve palsy (PNP) during right-sided pulmonary vein freezes (6, 7).

Diaphragmatic compound motor action potential (CMAP) monitoring during right phrenic nerve pacing was the first objective reported method dedicated to right phrenic nerve palsy (PNP) prevention. It was shown, first in animals and then in humans, that a 30% drop in CMAP amplitude (8) had to be considered as a threat to the right phrenic nerve. If this cut-off was reached, we showed that interruption of cryoapplication with forced deflation permitted to avoid a PNP.

The method for CMAP recording varies depending on local protocols. CMAP can be recorded with surface electrodes, for example by a modified lead I (9, 10); or *via* a quadripolar catheter positioned in a hepatic vein (11). The efficacy of both techniques has already been demonstrated separately (11–13) but they have never been compared. Our aim was to compare CMAP obtained with those two methods: first to evaluate the quality of signals at baseline, and then to evaluate surface CMAP evolution in case of right phrenic nerve threat seen on hepatic recording.

## Methods

### Study Design

A prospective single-center, comparative observational study was undertaken. Consecutive patients admitted to the local heart rhythm department for cryoballoon ablation were included if they were older than 18 years. AF ablation was offered according to current international guidelines (14). Patients with left atrial appendage thrombus on transoesphageal echocardiogram were excluded. Informed consent was obtained from the patients. Due to the retrospective nature of the study, with no active intervention, the study was deemed to be exempt by the institutional ethics review committee.

### Periprocedural Management and Cryoballoon Ablation Procedure

All patients underwent transesophageal and transthoracic echocardiography beforehand.

Procedures were performed with uninterrupted oral anticoagulation and under conscious sedation associated with local anesthetic. A quadripolar Josephson curve (Abbott, Lake Buff, IL, USA) catheter was positioned on the His bundle and a steerable quadripolar catheter with 4 mm electrodes and 2-5-2 spacing (Xtrem catheter; Sorin Group) was placed in the coronary sinus by femoral access. These catheters were used as landmarks to perform a single trans-septal puncture. Routine administration of unfractionated heparin was used with an initial bolus at 100 IU/kg and with a target activated clotting time of >300 ms.

The 15Fr steerable Flexcath sheath, 28 mm Arctic Front balloon (Medtronic Inc., Minneapolis, MN, USA) and Achieve Advance Mapping catheter (Medtronic Inc., Minneapolis, MN, USA) are employed for all cases. The objective was to achieve a single 180 s effective freeze for each pulmonary vein. A freeze was considered effective if either time to PVI was <60 s (if pulmonary vein signals were discernible on the Achieve Advance Mapping Catheter) or if the balloon temperature reached < -55°C. If neither criterion was satisfied, the freeze was maintained for 240 s. After each cryoapplication, PVI was assessed using the Achieve catheter. No extra freezes were routinely applied if definite isolation was achieved.

### Phrenic Nerve Monitoring

**Figure 1:**
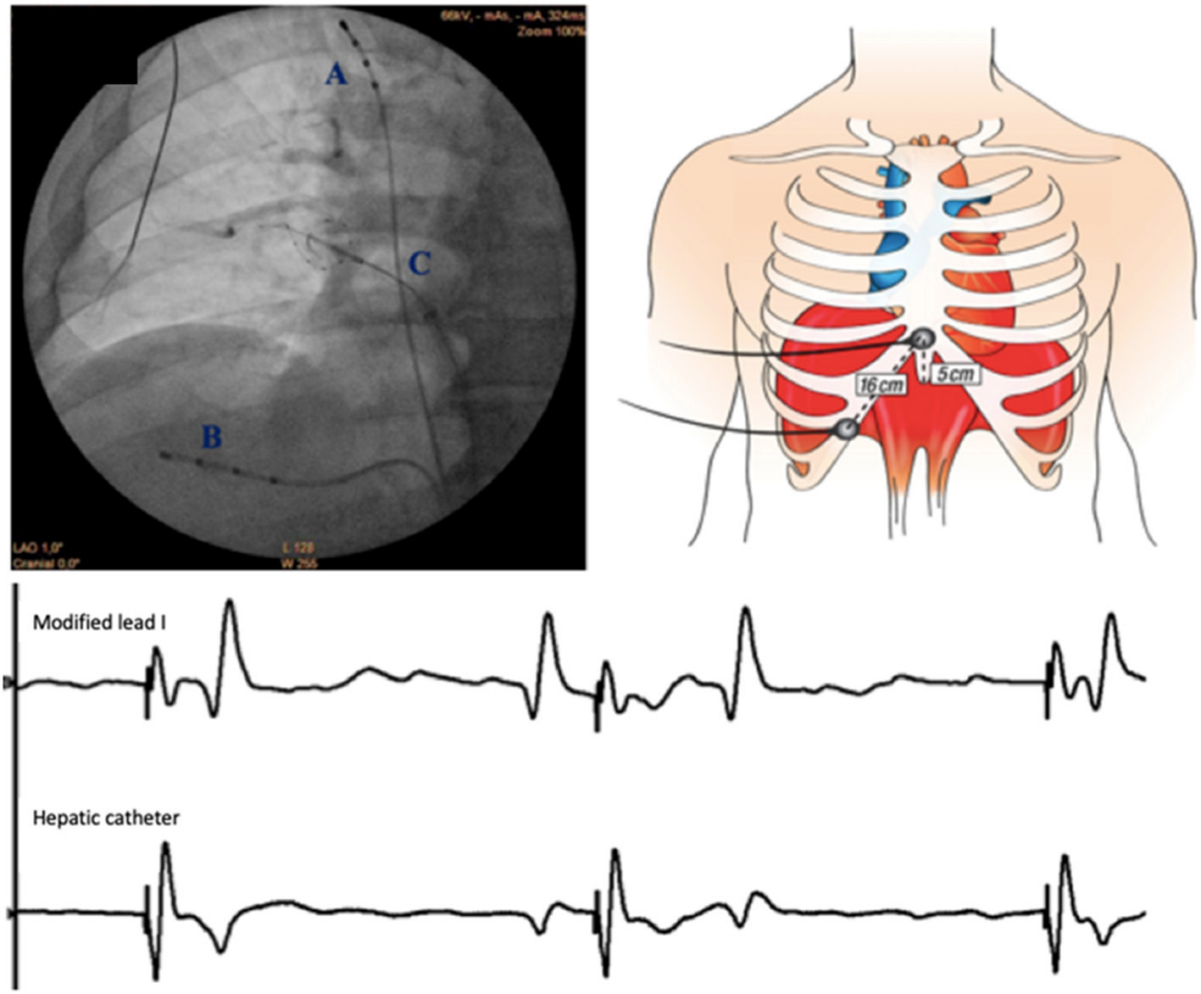
Set up for CMAP recording. *Left upper*: Catheters positions on an antero-posterior fluoroscopic view (A. pacing catheter in superior vena cava; B. recording quadripolar catheter in subdiaphragmatic hepatic vein; C. cryoballoon and Achieve catheter). *Right upper*: Configuration of surface electrodes to obtain modified lead I: the right arm electrode was placed 5 cm above the xyphoid process ant the left arm electrode along the right costal margin spaced 16 cm apart the xyphoid. *Lower*: simultaneous CMAP recordings with modified lead I and hepatic catheter. Sweep speed is 100 mm/s. The phrenic nerve is paced with a cycle length at 1,000 ms (60/min).

CMAP monitoring was performed using two methods (Figure 1). For the modified lead I ECG (10–15), signals were band-pass filtered between 0.5 and 40 Hz at the electrophysiology workstation. The quadripolar Hissian catheter was positioned into a subdiaphragmatic hepatic vein. Bipolar electromyographic signals were recorded between the proximal and the distal electrodes. Signals were amplified and band-pass filtered between 5 and 150 Hz.

During stage 1, after left-sided veins freezes, the steerable quadripolar catheter was relocated from the coronary sinus to the superior vena cava, to pace the right PN at 60 stimulations per minute (bipolar stimulation between proximal and distal electrodes with maximal output of 12V at 2.9 ms). If the obtained CMAPs remained steady, recordings were acquired simultaneously by both techniques for 1 m.

For stage 2, a real-time CMAP monitoring was performed during the right-sided freezes. If a phrenic threat was observed on hepatic signal (>30% drop in CMAP amplitude), application was immediately stopped using a forced deflation maneuver.

Unstable phrenic nerve capture was defined as a CMAP amplitude variation of >20% from beat-to-beat. Monitoring was judged not reliable in case great CMAP amplitude instability defined as CMAP amplitude variation >30% in more than 20 beats in a minute. A phrenic nerve threat was defined as a progressive amplitude drop of 30% within 10 s of onset. CMAP amplitude was monitored semi-quantitatively in a beat-to-beat analysis at the electrophysiology workstation by a trained physician.

### CMAP at Baseline

During first stage, CMAPs were compared in baseline conditions. The objective was to demonstrate which one was the easiest to interpret. We evaluated the ratio CMAP amplitude/ECG far-field amplitude. Then we compared CMAPs amplitude, stability (assessed beat to beat and after averaging 4 consecutives beats) and the number of uninterpretable signals during this minute of phrenic nerve pacing.

### CMAP at the Time of Right PN Threat

We assessed the signals in case of phrenic nerve threat (progressive amplitude drop >30% on hepatic signal) at the second stage of protocol. We evaluated surface CMAP evolution at the time of phrenic nerve threat diagnosed with a progressive drop in hepatic CMAP amplitude >30%.

Factors such as BMI, elevated abdominal perimeter, history of obstructive sleep apnoea or polypnea during the procedure were looked for as predictive of instable phrenic capture.

### Operator Experience

Seven electrophysiologists participated to this study. One was classified as an experienced operator because he had more than 10 years of experience with the cryoballoon and performed more than 50 procedures each year. The 6 others performed <30 procedures/year and had <5 years of experience with cryoballoon procedures.

### Statistical Analysis

Data are presented as a mean and standard deviation (SD) for continuous variables (if parametric), as a median with interquartile range (IQR) (non-parametric) and as numeric data with descriptive statistics for categorical variables. We compared the stability for each record method using the coefficient of variation which represents the ratio of the SD to the mean. This stability assessment was done beat per beat and using the average of four consecutive beats.

To compare the quantitative variables the tests used were Student's *t*-test and Wilcoxon's signed-rank test (if normality of data not known). A Chi-squared test was applied to categorical data. A *p*-value of <0.05 was considered to be statistically significant.

All authors had full access to and accept full responsibility for integrity of data.

## Results

### Patients and Procedures

Between March 2018 and February 2019, 118 patients underwent a cryoballoon ablation for AF. Four were excluded because of incomplete data: modified lead I not performed during the procedure; study not recorded at the electrophysiology workstation. Patients and procedure characteristics are summarized below (Table 1). Four complications occurred, including 3 instances of pericardial effusion (not requiring percutaneous drainage) and one pseudoaneurysm treated by prolonged extrinsic manual compression.

**Table 1:** Baseline patients characteristics.

**Patients**	**114**
Age, years, mean ± SD	61.7 ± 10.9
Sex, men (%)	77 (67.5)
BMI, kg/m^2^, mean ± SD	26.4 ± 3.8
Paroxysmal/persistent, %	55.3/44.7
History of diagnosis, months, mean ± SD	26.8 ± 31.5
EHRA 1/2/3, %	5.6/68.5/25.9
HTN/diabetes/OSA, %	36.2/8.8/11.4
Underlying cardiomyopathy (%)	46 (40.4)
Ischaemic Heart Disease (%)	17/46 (37.7)
CHADS VASC score, mean ± SD	1.63 ± 1.38
LVEF, %, mean ± SD	58.3 ± 11.3
LA volume index BSA, mL/m^2^, mean ± SD	39.7 ± 15.5
Length of hospital stay, days, mean ± SD	2.22 ± 0.73
Total procedure time, minutes, median (IQR)	72 (64–98)
Fluoroscopy time, minutes, median (IQR)	15 (11–27)
Radiation dose, μGym^2^, median (IQR)	2258 (1296–3763)
CB applications per vein, number, mean ± SD	1.42 ± 0.79
Cryotime for each vein, seconds, mean ± SD	280 ± 134
Lowest temperature, degree, mean ± SD	−48.4 ± 8.6
Time before PVI, seconds, mean ± SD	45.6 ± 29.7

*BMI, body mass index; BSA, body surface area; CB, cryoballoon; EHRA, European Heart Rhythm Association; HTN, arterial hypertension; LA, left atrium; LCT, left common trunk; LVEF, left ventricular ejection fraction; OSA, obstructive sleep apnoea; PV, pulmonary vein; PVI, pulmonary vein isolation; RCT, right common trunk; RMPV, right middle pulmonary vein; SD, standard deviation*.

### Stage 1: Baseline Phrenic CMAP Recording

To obtain a steady baseline phrenic CMAP signal (amplitude at least 0.30 mV), the hepatic catheter was re-positioned in 34/114 patients (29.8%). The average number of attempts to achieve a satisfactory position was 1.53 (±1.03). Median time to final satisfactory position was 26 s and median additional fluoroscopy time was 20 s. Phrenic capture was considered unstable for 43% of patients. CMAP monitoring technique was not reliable because of pacing instability in 15 patients (13.16%). There were no factors predictive of this phenomenon.

**Figure 2:**
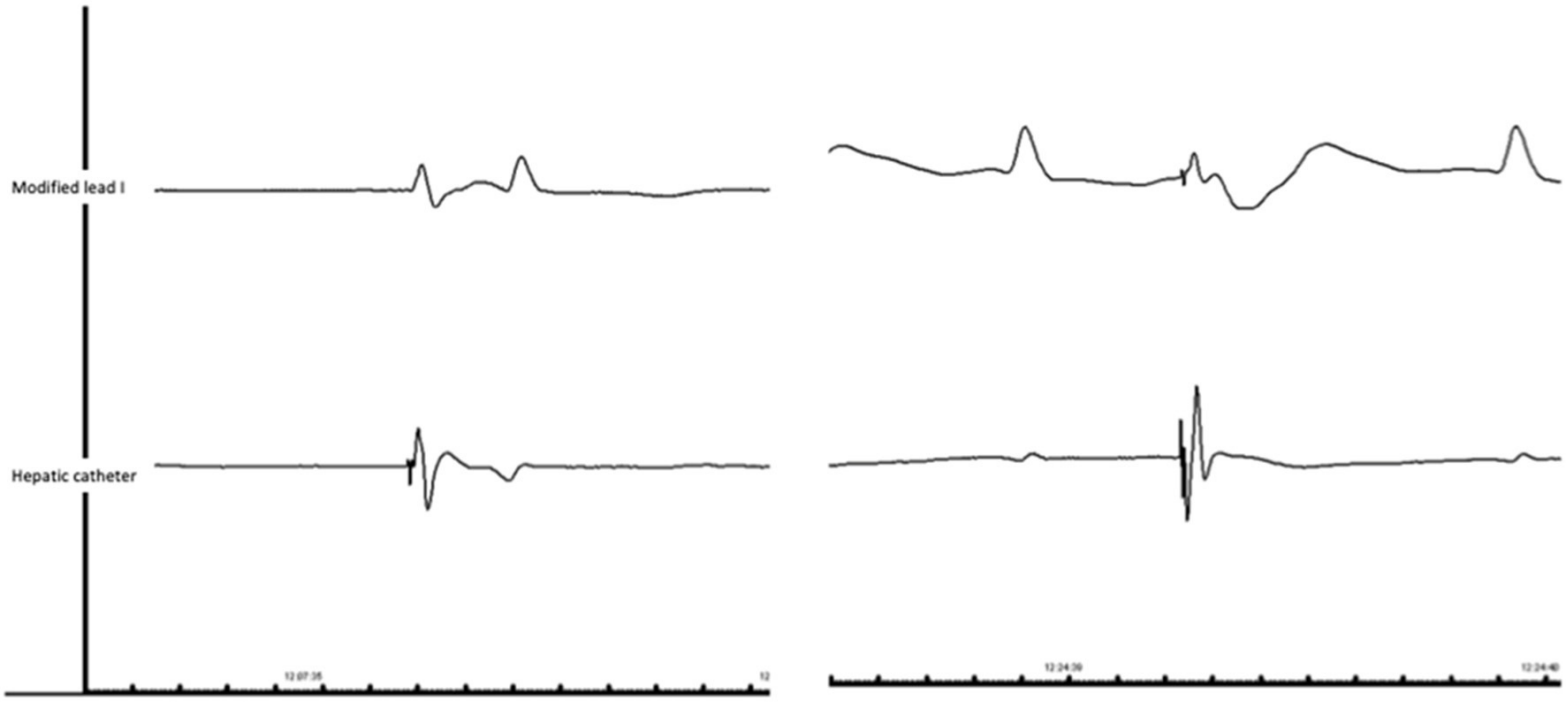
2 most common patterns of CMAP in our population. *Left*: First pattern: biphasic signal recorded in surface electrode with CMAP/QRS ratio about 1; biphasic and sharp signal recorded with hepatic catheter (Patient#26). *Right*: Second pattern: monophasic and slurred signal recorded in surface electrode with CMAP/QRS ratio inferior to 1; sharp triphasic signal recorded with hepatic catheter (Patient#19).

CMAP morphology varied by method, with sharp signals for hepatic recording and slurred and often monophasic signals for the modified lead I (Figure 2). The stage 1 outcomes results are presented below (Table 2).

**Table 2:** Primary and secondary outcomes of stage 1: basal CMAP recording during 60 s before any right-sided cryoapplication.

	**Hepatic record**	**Surface record**	***p*-value**
Ratio CMAP/QRS, median (IQR)	4.63 (2.67–9.46)	0.76 (0.55–1.14)	<0.0001
Coefficient of variation, beat to beat, median (IQR)	7.83 95.54–10.85)	7.43 (5.27–10.27)	0.3114
Coefficient of variation, averaged over 4 consecutive beats, median (IQR)	3.92 (2.48–6.74)	4.10 (2.85–5.96)	0.2177
Amplitude, mV, median (IQR)	0.48 (0.31–0.82)	0.89 (0.72–1.10)	<0.0001
Uninterpretable signals/60, *n*, median (IQR)	0 (0–1.67)	10 (5–13.33)	<0.0001

*IQR, interquartile range*.

### Stage 2: Right Sided PV Ablation With Phrenic Nerve Monitoring

A total of 19 forced deflation maneuvers were performed. In 5 cases, there was no phrenic threat but rather phrenic nerve capture instability.

There were 14 phrenic threats, occurring in 12/114 patients (10.5%): 10 during RUPV freeze and 4 during RLPV freeze (Table 3).

**Table 3:** All patients (12) with phrenic threats (14) during cryoballoon ablation: demographic and technical data.

**Patient**	**Sex** **and age**	**Stable** **pacing**	**Location**	**Freeze/total** **freezes**	**Minimum** **temperature (°C)**	**Application** **duration** **(sec)**	**Late forced** **deflation** **(sec)**	**Diaphragm** **contractility** **reduction**	**CMAP amplitude** **recovery**
#10	M, 64	No	RUPV	1/1	−50	65	44	Yes	No
#19	M, 41	Yes	RUPV	1/2	−48	77	9	No	Total
#19	M, 41	Yes	RUPV	2/2	−6	90	3	No	Total
#22	F, 71	Yes	RLPV	1/3	−37	80	3	No	Total
#25	M, 69	Yes	RLPV	1/1	−52	165	6	No	Total
#30	F, 70	No	RUPV	1/1	−52	141	36	Yes	Partial
#38	F, 69	No	RUPV	1/2	−41	75	13	No	Total
#38	F, 69	No	RUPV	2/2	−44	78	3	No	Total
#61	F, 54	No	RLPV	1/2	−65	52	8	No	Total
#75	F, 72	Yes	RUPV	1/2	−47	112	3	No	Total
#81	M, 63	No	RUPV	1/1	−61	172	*MV*	No	Total
#84	M, 61	Yes	RUPV	1/1	−52	204	13	No	Partial
#89	M, 48	No	RLPV	1/1	−53	162	14	No	Total
#112	M, 66	No	RUPV	1/1	−54	166	41	No	Partial

*F, female; M, male; MV, missing value; RLPV, right lower pulmonary vein; RUPV, right upper pulmonary vein*.

At the precise time defined as phrenic threat, there was an amplitude drop of 35.61 ± 8.27 % in the hepatic arm vs. 33.42 ± 11.58 % in the surface arm (*p* = 0.54).

Overall, median time to forced deflation once a 30% CMAP drop was detected was 9 s (3–14) s. In the 4 patients (3.51%) with partial or no recovery, median time to forced deflation was significantly longer at 38.5 s (30.3–41.8) (*p* = 0.03). Time to deflation was 44 s in the only patient with persistent PNP.

Pulmonary vein disconnection was successful in 100% of cases, despite shortened cryoapplication. In 4 patients, a second application was performed after the phrenic threat, and in one patient 2 additional freezes were undertaken. The average delay before phrenic threat was 98.0 ± 48.0 s after cryoablation commencement.

## Discussion

To our knowledge, this is the first study comparing two phrenic CMAP recording methods to prevent phrenic nerve palsy during cryoballoon ablation for atrial fibrillation.

Thus, far, all prior neurological studies investigating phrenic CMAP monitoring have used surface recording as the gold-standard method. Hepatic recording had previously only been used during cardiac electrophysiological procedures. Good correlation between phrenic nerve signals with both detection methods has been demonstrated. Hence, this alternative option for diaphragmatic electromyography recording could be used for multiple other applications for example in diagnosis and management of neuromuscular diseases.

Phrenic nerve monitoring using a hepatic catheter therefore seemed feasible, and the additional procedure duration and fluoroscopy times were only modest. Nevertheless, any change in procedural protocols can result in an increased learning curve. Thus, the most experienced operator needed 27.3 ± 27 s extra fluoroscopic time until obtaining a satisfactory catheter position vs. 52.0 ± 64.7 s for the others (*p* = 0.01).

At baseline, the absolute CMAP amplitude was significantly higher on surface recording. The ratio of CMAP/QRS with the hepatic catheter was seven-fold greater than the ratio on surface. Consequently, in the case of fusion of CMAP and far-field QRS (or more rarely far-field T waves), the signal could still be interpreted easily.

Amplitude variations observed with the hepatic method were similar to those with surface recording. Clearly, modified lead I could also be used but we suppose that better legibility (sharper signals, low amplitude of recorded far-field QRS) made minor variations more apparent in real-time with the hepatic monitoring method.

Phrenic nerve pacing can be achieved with different types of catheters. We used a quadripolar deflectable Xtrem Catheter (Microport). The electrodes size is 4 mm and the inter electrode space is 2–5-2 mm. Pacing was performed between electrode 1 and 4, so the dipole size was 25 mm. We don't believe that pacing stability may have been improved with a decapolar catheter. First, electrodes size in decapolar catheters is usually 2 mm. One could hypothesized that decreasing the size of the electrodes can decrease the amount of energy delivered to the nerve. Second, even with a decapolar catheter, the pacing is usually delivered between electrode 1 and 4. The dipole size in that case can even be reduced because of the reduction of the electrodes size. To our knowledge, there is no data concerning a higher phrenic nerve pacing stability with decapolar catheters.

Pacing instability during monitoring made amplitude drops difficult to interpret (5 inappropriate freeze terminations). Failure to achieve stable phrenic nerve capture was observed in 43% of cases which was relatively high when compared to previous series (13). This was mainly due to the significant variability of electrophysiologists' experience. Indeed the rate of unstable capture varied between 31.7 and 73.3% according to physician. Difficulties experienced in achieving stable capture might be also explained by catheter choice. Unfortunately, there is no specific catheter for phrenic nerve stimulation so far.

The prevalence of phrenic threats in our series was 10.5%, slightly lower than the 12–14% reported previously (11, 13). The low event rate of this complication makes the small differences between studies difficult to interpret.

Freeze termination was not systematically performed at the point of 30% drop in amplitude, as is recommended. Further, in the cases with longest time to freeze termination, there was a greater prevalence of incomplete phrenic nerve recovery. In light of this, an automated reading system is paramount to diagnose a phrenic threat as early as possible, especially if a surface monitoring is chosen (slurred signals, more artifact due to far-field QRS).

The main limitation of this study is that it is observational in nature. Both methods were utilized for each patient to allow comparison of signal acquisition. It is now essential to compare, in a randomized fashion, to assess the ability of each method to detect phrenic threat early and prevent phrenic nerve palsy.

## Conclusion

This study confirms phrenic CMAP monitoring during cryoballoon ablation is clinically feasible using either ECG surface electrodes or a diagnostic catheter in a hepatic vein. Although stability is similar with both methods, signal clarity and quality are much better with hepatic recording. This may allow the operator to detect significant drops in amplitude sooner, enabling them to discontinue cryoapplication before phrenic nerve injury occurs. The technique remains slightly limited by operator inexperience, as evidenced by variability in achievement of stable phrenic signal capture. Despite this, using appropriate technologies in the future such as a specific phrenic nerve pacing catheters or automated CMAP amplitude reading systems, it may be possible to markedly improve inter-operator variability.

## Data Availability Statement

The raw data supporting the conclusions of this article will be made available by the authors, without undue reservation.

## Ethics Statement

The studies involving human participants were reviewed and approved by Ethic Commitee of Aix Marseille Université. The patients/participants provided their written informed consent to participate in this study.

## Author Contributions

FF and LT: conceived, designed, and performed the analysis and wrote the paper. FF, LT, BM, LK, JH, EM, MZ, and J-CD: collected the data. FF, LT, BM, JH, EM, MZ, and J-CD: contributed data or analysis tools. All authors contributed to the article and approved the submitted version.

## Conflict of Interest

The authors declare that the research was conducted in the absence of any commercial or financial relationships that could be construed as a potential conflict of interest.

## Publisher's Note

All claims expressed in this article are solely those of the authors and do not necessarily represent those of their affiliated organizations, or those of the publisher, the editors and the reviewers. Any product that may be evaluated in this article, or claim that may be made by its manufacturer, is not guaranteed or endorsed by the publisher.
